# Step Away from Depression—Study protocol for a multicenter randomized clinical trial for a pedometer intervention during and after in‐patient treatment of depression

**DOI:** 10.1002/mpr.1862

**Published:** 2020-11-12

**Authors:** Julia Große, Moritz Bruno Petzold, Ralf Brand, Andreas Ströhle

**Affiliations:** ^1^ Klinik für Psychiatrie und Psychotherapie Campus Charité Mitte Charité—Universitätsmedizin Berlin, corporate member of Freie Universität Berlin, Humboldt‐Universität zu Berlin, and Berlin Institute of Health Berlin Germany; ^2^ Sport and Exercise Psychology University of Potsdam Potsdam Germany

**Keywords:** exercise, mental disorder, MoVo, steps

## Abstract

**Objectives:**

Increasing the number of daily steps by using a pedometer and a diary leads to an activity increase and improved health outcomes in a variety of somatic disorders. Hence, for the inpatient treatment of depression, supervised exercise interventions are more widespread. We aim to examine if a self‐managed pedometer intervention (PI) with the option of being proceeded after discharge leads to reduction of depression and to a physical activity (PA) increase.

**Methods:**

The Step Away from Depression (SAD) study is a multicenter randomized controlled trial targeting 400 patients with major depressive disorder. Treatment as usual (TAU) is compared to TAU plus PI after 4 weeks, at discharge, and 6 months after hospital admission. Primary outcomes are clinically rated depression severity and accelerometer‐measured step counts. Secondary outcomes include self‐reported depression symptoms and PA level, psychiatric symptoms, health‐related quality of life, self‐efficacy, and components of the Motivation Volition Process Model.

**Results:**

We report the design of the SAD study considering several methodological aspects for exercise studies, in general.

**Conclusions:**

Results of our study will provide information about efficacy of PI for inpatient treatment and about interrelating processes of change concerning depression, PA, and aspects of motivation and volition.

## 1 INTRODUCTION

### 1.1 Physical activity and depression

Depressive disorders are the “largest contributor to nonfatal health loss” (World Health Organization, 2017) worldwide and show a global point prevalence rate of 4.7% (Ferrari et al., 2013). Furthermore, patients with depression are significantly more prone to suffer from somatic disorders, especially chronic diseases (Lotfaliany et al., 2018; Moussavi et al., 2007; Steffen, Nübel, Jacobi, Bätzing, & Holstiege, 2020). Therefore, the effective and long‐lasting treatment of depression remains one of the most challenging issues in mental health care. At present, the German S‐3‐guidelines recommend psychotherapy and/or, according to severity of symptoms, psychopharmacotherapy (DGPPN, 2015). Likewise, from a good clinical practice point, they recommend regular physical activity (PA). Besides therapeutical effects, PA also shows a preventive influence on depression with an adjusted odds ratio of 0.83 (95% CI = 0.79, 0.88; *I* ^2^ = 0.00) for people with higher PA compared to those with lower PA (F. B. Schuch et al., 2018).

PA has been increasingly investigated during the last years with reference to its effectiveness in the treatment of depression. Mutrie, Richards, Lawrie, and Mead (2018) give a detailed overview about current research on this topic. Several meta‐analyses (Cooney et al., 2013; Kvam, Kleppe, Nordhus, & Hovland, 2016; Miller et al., 2020) confirm a small to moderate effect (with effect sizes of *d* = −0,31 up to −0,68) of exercise as a treatment of depression. However, even large effects are demonstrated, showing that underestimation of the exercise‐depression‐effect in the former meta‐analyses may be possible (Ekkekakis, 2015; F. B. Schuch et al., 2016).

### 1.2 Daily amount of PA in healthy people and in patients with depression

The World Health Organization (WHO) proposes an amount of 150 min of moderate activity or 75 min of vigorous activity per week or any corresponding combination of these two (World Health Organization, 2010). Moreover, this activity should be executed in time bouts of at least 10 min 63.2% of the overall population of high‐income Western countries comply with these PA sufficiency recommendations (Guthold, Stevens, Riley, & Bull, 2018). Reaching the goal of 10,000 steps per day is still a popular goal in pedometer use (Tudor‐Locke et al., 2011) Healthy people aged 18–65 years usually walk 9797 steps per day (Bohannon, 2007). However, especially concerning low active persons, raising the amount of steps gradually and regularly seems to be more important for health benefit than reaching the goal of 10,000 steps per day (Hallam, Bilsborough, & de Courten, 2018; Lee et al., 2019). Of people with mental disorders, 41.7% fulfill the criteria of the American College of Sports Medicine for PA (30 min of moderate activity on 5 days or 20 min of vigorous activity on three days or 1000 MET minutes in 1 week; Petzold et al., 2017). The percentage of people with depression with insufficient PA seems to be roughly twice as high as that of healthy individuals. Around two thirds (67.8 %) of depressive patients report insufficient PA (F. Schuch et al., 2017). Overall, literature shows that PA self‐report data likely overestimates objective PA (Chaudhury, Stamatakis, Roth, & Mindell, 2010; Garriguet, Tremblay, & Colley, 2015). When only objective measures of PA are taken into account, the percentage of insufficiently active depressive patients even increases up to 85.7. In summary, the figures are alarming and call for interventions to increase daily life PA, even more for people with depressive disorders.

### 1.3 Walking as a PA intervention

Pursuing the wider aim of implementing PA interventions in routine care, the following characteristics appear to be reasonable: First, PA interventions have to be easily applicable for patients in an inpatient and outpatient treatment and in a self‐manage setting. Second, taking into account the restriction of financial and human resources in health care as well as the loss of motivation and self‐efficacy that many patients with depression show, exercise interventions for depression should be as simple as possible. Third, the higher prevalence of somatic disorders in patients with depression requires an individual adaptivity concerning the intensity and frequency of PA. In a questionnaire study, depressive patients show a preference for a flexible walking intervention over nine other types of PA like weight lifting, yoga, and dance (Busch et al., 2016). A large and significant effect with a pooled standardized mean difference of −0.86 (with CI of −1.12 to −0.61) compared to control group is shown for walking interventions for depression (Robertson, Robertson, Jepson, & Maxwell, 2012).

One simple and effective tool for increasing walking activity are pedometers. This more objective measure of PA may help to overcome the problem of biased self‐report (Bravata et al., 2007; Tudor‐Locke, Williams, Reis, & Pluto, 2002). The use of pedometers is associated with an increase in PA in healthy subjects, in patients with physical illness or psychiatric diseases (Bravata et al., 2007). Depressive symptoms can be decreased by pedometer use (Abedi, Nikkhah, & Najar, 2015; Piette et al., 2011). Low financial, time, and personnel costs of pedometer interventions (PIs) are important additional reasons to pursue the implementation of this intervention. However, adequately powered randomized controlled clinical trials in patients with major depression are not available in the literature.

### 1.4 Methodological aspects of PA studies and their consideration in SAD

#### 1.4.1 Collecting data of daily PA

Although studies with PA interventions for depression do report clinical changes, they often fail to do so for a possible change of PA in the patients (Balchin, Linde, Blackhurst, Rauch, & Schonbachler, 2016; Piette et al., 2011). Sometimes, adherence rates to the intervention program are given (Kerling et al., 2015; F. B. Schuch et al., 2015), indicators of body functioning/fitness (e.g., timed walk tests) are presented (Heinzel, Lawrence, Kallies, Rapp, & Heissel, 2015) or weekly feedback is collected (Kerr et al., 2008). It is essential to know whether patients with additional PA interventions move more than patients with treatment as usual (TAU) but with an equal symptom severity of depression after treatment ending.

#### 1.4.2 Combination treatment

More research on combination treatments with TAU and TAU + exercise is needed because present findings are not clear concerning the effect size, so far (it has a range of *g* = −0.68 to *g* = −0.08; Kvam et al., 2016). This is mostly due to differences in methodology of the studies and in program variables of the exercise interventions. The effect of exercise decreases with blinded outcome, intent‐to‐treat‐analysis and when compared to guideline TAU.

#### 1.4.3 Follow‐up

Many treatments lose their effect after treatment ended and are no longer effective at follow‐up (Cooney et al., 2013; Kvam et al., 2016). For this reason, interventions that are applicable to in‐patient treatment as well as to following self‐management (often in combination with outpatient care) are necessary. Thus, collecting follow‐up data in PA trials is of immense importance.

#### 1.4.4 Employing psychological theories

PA interventions which incorporate theories of exercise psychology lead to better results (Thomas, Thirlaway, Bowes, & Meyers, 2020). For this reason, we use the Motivation Volition Process Model (MoVo‐Model; Fuchs, 2007). Whereas many psychological theories of behavior change focus on motivational variables, the MoVo‐Model includes additional postintentional variables. It splits the behavior change process into two phases—the motivational phase (with components like self‐efficacy, outcome expectations, intention strength, and self‐concordance) and the volitional phase (with the components of action planning, barrier management and situational cues). First studies using MoVo‐Model in populations with mental disorders show that the model might be as helpful for the design of interventions in this population as in populations without mental disorders (Petzold et al., 2017). Interventions built on this model lead to promising effects in patients with mental disorders (Gohner, Dietsche, & Fuchs, 2015; Petzold et al., 2019).

#### 1.4.5 General methodological aspects

Further methodological aspects such as blindedness of outcome ratings, taking into account anxiety as the most frequent comorbidity of depression and the applicability to real‐life clinical settings were insufficiently put into practice in many former studies (Bond, Stanton, Wintour, Rosenbaum, & Rebar, 2020; Cooney et al., 2013; Kvam et al., 2016). Thus, the need for studies considering these aspects still exists. For the individual patient, even a mildly increased effectiveness of their depression treatment could make a big difference, especially in those with residual symptoms. If successful, our study would give strong empirical support for the use of pedometers to increase PA in patients with major depression during and after inpatient treatment.

### 1.5 Rationale and objective

#### 1.5.1 Primary objective

Our **primary objective** is to determine whether the use of a pedometer and an activity book incorporating strategies of self‐monitoring and goal setting can help in‐patients with major depression to increase steps and to further decrease depression in comparison to TAU.

#### 1.5.2 Secondary objectives

**Most important secondary objectives** are to compare changes in subjective PA, objective PA, anxiety severity as well as in the duration of in‐patient treatment between PI and TAU.

Further **secondary objectives** are to determine if the PI group differs from TAU group from baseline (first to third day of inpatient treatment) to end of inpatient treatment (three days before end of inpatient treatment) in

1. psychopathological symptoms
2. health‐related quality of life
3. self‐reported depressive symptoms
4. PA: self‐efficacy, intention, self‐concordance of goal‐striving, outcome expectations, planning, and barrier planning
5. general self‐efficacy

#### 1.5.3 Research hypotheses

Hypotheses of this trial are:

From hospital admission to the end of inpatient treatment patients of the PI group in contrast to the TAU group show:

1. Increased PA and decreased depression
2. Decreased anxiety and duration of hospital treatment

## 2 METHOD

### 2.1 Study design

**FIGURE 1:**
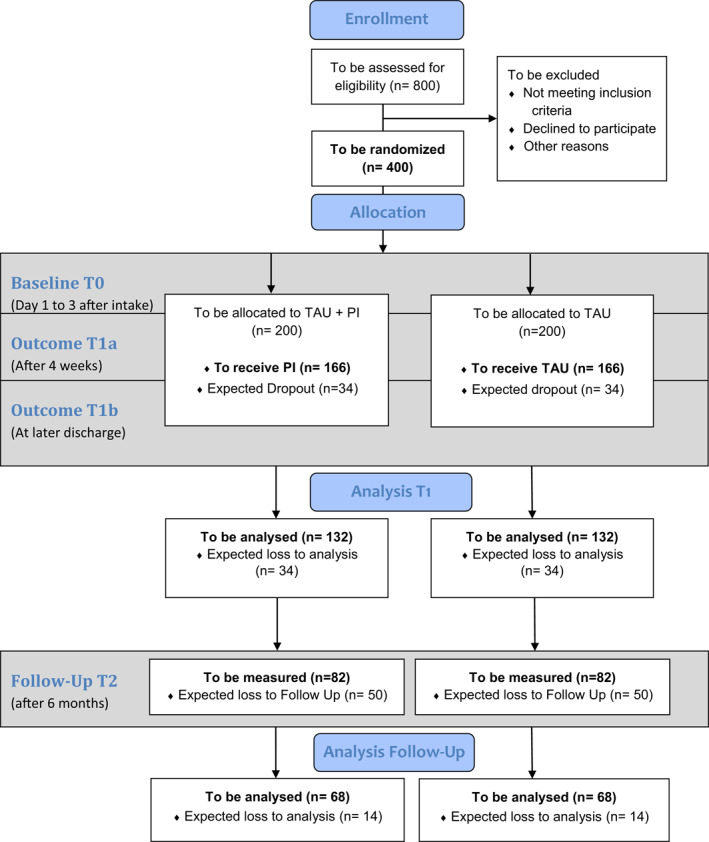
Flowchart of the SAD study. PI, pedometer intervention; SAD, Step Away from Depression; TAU, treatment as usual

We use two treatment arms in a longitudinal randomized single‐blinded (rater)controlled parallel group design with points of measurement at Day 1–3 after hospital admission (baseline, T0), after 4 weeks (T1a), at discharge (T1b), and 6 months after hospital admission (follow‐up, T2; see Figure 1).

### 2.2 Study setting

Selection of these clinics is done due to collaboration in the “Referat für Sportpsychiatrie und –psychotherapie” of the DGPPN including universitary as well as municipal psychiatric clinics in urban and rural areas.

### 2.3 Participants and eligibility

#### 2.3.1 Inclusion criteria

Inpatients are eligible to the trial if they

1. have unipolar major depression as primary diagnosis
2. are aged from 18 to 65 years
3. have a prospected inpatient treatment of at least 4 weeks
4. are able to understand German language and
5. can be recruited within the first three days after hospital admission

#### 2.3.2 Exclusion criteria

Exclusion diagnoses are psychotic depression, borderline personality disorder, schizophrenia, anorexia nervosa, and dementia. Furthermore, we exclude patients with current substance addiction (besides nicotine), acute suicidality, pregnancy, regular use of pedometers, and patients with medical disorders resulting in the inability to walk with mild to moderate intensity at least 5000 steps per day or physical, laboratory, or electrocardiography findings that would put patients at risk if they increase their daily steps. Patients with more than 10,000 steps at baseline are equally excluded from the study.

### 2.4 Randomization and treatment conditions

#### 2.4.1 Randomization

Patients are randomly allocated into one of two groups: (1) TAU or (2) TAU plus PI. Randomization—done by a computerized random number generator—is stratified by center with an intended allocation ratio of 1:1. Group assignment is implemented via sequentially numbered, sealed, opaque envelopes. Group allocation is disclosed after baseline measurement.

#### 2.4.2 Treatment as usual

TAU, the control condition, comprises inpatient clinical treatment with psychotherapy in group and/or individual settings as well as pharmacotherapy and other adjuvant treatments like sociotherapy, occupational therapy, music therapy, physiotherapy. Most clinics also offer PA interventions such as exercise therapy or yoga. Concomitant care is not restrained in both groups during and after inpatient treatment. Patients in the TAU condition do not receive a pedometer or diary.

#### 2.4.3 Pedometer intervention

**FIGURE 2:**
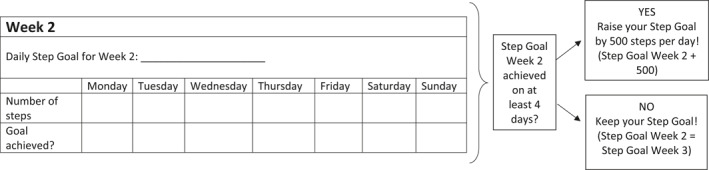
Example of “Week 2” of the activity diary

Patients in PI group receive an Omron Walking style IV pedometer (Model HJ‐325‐EW)—a small tool, fixed at the belt or worn in the pockets that records number of steps. Additionally, we provide patients with an activity book. Instruction is to fill in daily steps as displayed by the pedometers. Furthermore, patients should increase the daily step goal by 500 steps compared to their former base level (see Figure 2). Pedometers are blinded by masking their display with adhesive tape for baseline measurement. The average step number of these three days serves as starting value for the intervention. By the beginning of the PI pedometers are unblinded. If patients achieve the new goal for at least 4 of 7 weekdays, they should continue increasing the number of steps for the next week. If they do not reach their goal, they should keep this goal again for the coming week. When 10,000 steps per day are attained, patients can individually decide whether to further increase the level or to continue with 10,000 steps. To sum up, PI consists of (1) daily wearing of the pedometer, (2) recording steps in the activity diary, and (3) weekly raising number of steps by 500 or maintaining 10,000 daily steps, during the whole inpatient‐stay and afterward for a total of 26 weeks.

### 2.5 Procedure and recruitment

Trial staff members in different centers are psychiatrists, clinical psychologists, and/or advanced students of psychology or medicine. All staff members are trained with standard material and instructions. Main task of the staff is to carry out measurements in a uniform reproducible manner and to give predefined instructions to the PI group how to use the pedometer and the activity diary. Participants fill out questionnaires online via SoSci‐Survey (Leiner, 2019) under www.soscisurvey.de or, if not possible (due to technical restrictions of the clinic or personal preferences of the patient), by paper–pencil.

Recruitment takes place at hospital admission or up to 3 working days after. Eligibility of patients is checked systematically for every patient newly admitted to the ward. The study personnel contacts eligible patients, delivers information sheets about the study and gives opportunity to discuss any belonging issue. In case of acceptance, patients give their written informed consent. Trial staff members then arrange all measurement appointments with patients throughout the study.

To promote retention, patients receive reimbursement of 15 Euro only if they attend the Follow‐Up‐Measurement. Patients are not controlled how they follow the PI or how pedometer data is recorded in the diary. In order to monitor adherence of the PI group to the intervention, study personnel makes copies of the activity diary at T1‐ and T2‐Measurement. PI participants are allowed to keep their pedometer for personal use after participation in the study, independently of adherence or dropout.

### 2.6 Data collection

Table 1 displays all measurements at different points in time line. All questionnaires are administered in German language.

**TABLE 1:** Time table of measurements

		T0 (baseline)	T1a (4 weeks)	T1b (discharge)	T2 (follow‐up)
Physical activity					
Objective	Accelerometer measurement for 3 days (primary outcome)	x	x	x	x
Subjective	**Q:** IPAQ short version (7)	x	x	x	x
Adherence	**AD:** Copy of step protocols (IG only)	‐	x	x	x
Depression					
Objective	**I:** Montgomery–Åsberg Depression Rating Scale (primary outcome) (10)	x	x	x	x
Subjective	**Q:** Beck‐Depression‐Inventory II (21)	x	x	x	x
General, physical, and clinical measures					
General measures	**Q:** Sociodemographic data, medication	x	‐	‐	‐
Physiological measures	**HR:** Blood pressure, heart rate, weight, and blood glucose	x	‐	‐	‐
Laboratory values	**HR:** Triglycerides, cholesterol, high density lipoprotein, low density lipoprotein	x	‐	‐	‐
Clinical history	**Q:** Anamnestic questions (6)	x	‐	‐	‐
Psychiatric symptoms	**Q:** Symptom checklist 27 (27)	x	x	x	x
Anxiety	**Q:** Beck anxiety inventory (21)	x	x	x	x
General health quality	**Q:** Short‐Form Health‐Survey 12 (12)	x	x	x	x
Intervention evaluation	**Q:** Questions about the implementation of PI (5)	‐	‐	‐	x
Treatment history	**Q:** Concomitant treatment since discharge (13)	‐	‐	‐	x
Critical life events	**Q:** Social Readjustment Rating Scale (43)	‐	‐	‐	x
Components of the MoVo‐model					
Intention, motivation and volition	**Q:** Intention (2), physical activity specific self‐efficacy (6), outcome expectancies (6), self‐concordance scale (12), action planning (4), coping planning (4)	x	x	x	x

Abbreviations: ( ), number of items; AD, activity diary; HR, information out of hospital record; I, interview; IPAQ, International Physical Activity Questionnaire; MoVo‐Model, Motivation Volition Process Model; PI, pedometer intervention; Q, Online‐Questionnaire or Paper/Pencil.

### 2.7 Primary outcome measures

#### 2.7.1 PA—Objective

To objectively measure steps and overall PA we use ActiGraph GT1M accelerometers (ActiGraph). The GT1M counts the incidences of change in acceleration per minute and can therefore measure the amount of PA. Additionally, it measures steps. Accelerometers have already been used in former research and proved being a reliable instrument for measuring PA (Kaminsky & Ozemek, 2012; Silva, Mota, Esliger, & Welk, 2010), also for samples with patients with mental disorders (Petzold et al., 2017). For different activity levels, we set thresholds according to the ActiLife Software (ActiLife 6 User's Manual, 2008): light (≤1952 counts per minute), moderate (1953–5724 counts per minute), hard (5725–9498 counts per minute), and very hard (≥9499 counts per minute). For measuring the activity of less active people, two to three valid days of wearing the accelerometer are enough for sufficiently valid data (Kocherginsky, Huisingh‐Scheetz, Dale, Lauderdale, & Waite, 2017). Therefore, patients are instructed to carry the accelerometer around their hip for 3 consecutive days while being awake. Mean step count is used as summary measure format.

#### 2.7.2 Depression—Objective

For objective rating we use the Montgomery–Åsberg Depression Rating Scale (MADRS; Montgomery & Asberg, 1979). Scores of 10 or less on the MADRS are considered to indicate remission (Hawley, Gale, & Sivakumaran, 2002; Zimmerman, Posternak, & Chelminski, 2004) and a decrease of 50% of symptoms as response. MADRS assessments are performed by blinded staff personnel. Raters are trained to ensure interrater reliability. Interrater correlations of these trainings show a Cronbach's Alpha of 0.978 (CI: 0.95–0.99).

### 2.8 Secondary outcome measures

#### 2.8.1 PA—Subjective

Self‐reports of PA are assessed with the International Physical Activity Questionnaire (IPAQ; Craig et al., 2003). We use the short “last 7 days” self‐administered version measuring time spent with four activity levels (vigorous activity, moderate activity, walking, and sitting). The IPAQ correlates moderately with accelerometer data in samples with mental disorders (overall PA: *r* = 0.47; Petzold et al., 2017).

#### 2.8.2 Depression—Subjective

Beck Depression Inventory II (Beck, Steer, & Brown, 1996) measures depression subjectively.

#### 2.8.3 Anxiety

**Anxiety** symptoms are measured with the Beck Anxiety Inventory (Beck, Epstein, Brown, & Steer, 1988).

#### 2.8.4 Other measures

We collect data on sociodemographic variables (sex, age, educational level), former depression episodes and treatments and former or current somatic diseases of the patient. Furthermore, **psychiatric symptoms** are measured using the Symptom‐Checklist‐27 (Hardt & Gerbershagen, 2001) and **health‐related quality of life** with the 12‐Item Short‐Form Health Survey (Ware, Kosinski, & Keller, 1996). As physiological parameters, we collect blood pressure, heart rate, weight, blood glucose, and laboratory values of triglycerides, cholesterol, high density lipoprotein and low density lipoprotein. Because these parameters are not measured routinely in clinics at discharge, only baseline data will be available.

#### 2.8.5 Motivational and volitional factors of the MoVo

Besides, we aim to explore the motivational and volitional determinants of PA from the MoVo‐Model.

**Specific self‐efficacy** is measured with six items according to earlier research (Schwarzer, Lippke, & Luszczynska, 2011). There are two items each for action self‐efficacy, maintenance self‐efficacy, and recovery self‐efficacy.

The other constructs are measured according to scales used by Petzold et al. (2017), providing a more detailed description of these measures. The scales contain items about **intention**, **outcome expectancies**, **action planning,** c**oping planning**, **self‐concordance,** and **general self‐efficacy**.

### 2.9 Data analyses

#### 2.9.1 Calculation of power and sample size

*For depressive symptoms,* Cooney et al. (2013) report a large effect size of −1.22 (−2.21, −0.23) in favor for exercise plus treatment versus treatment, and a medium effect size for all control conditions with *d* = −0.62. For the subgroup of light/moderate activity interventions, they report an also large effect size of −0.83 (−1.32, −0.34). Therefore, we also expect a medium effect size in the MADRS between both groups at discharge. *For PA,* Heinzel et al. (2015) find an effect size of *d* = −0.49 (95% CI −0.91 to −0.06, *Z* = 2.23, *p* = 0.03) in favor of exercise for body functioning/fitness indicators in five trials of their meta‐analysis. Thus, we expect a small but close to moderate effect size for PA difference at discharge.

Taken together, we assume a small to moderate effect size for the group difference. According to Cohen (1988), *f* ^2^ = 0.02 and *f* ^2^ = 0.15 represent small and moderate effect sizes, respectively. Conservatively, we aim to detect a small effect of *f* ^2^ = 0.06. For this, in a parallel group, fixed sample trial with a multivariate analysis of variance (MANOVA; 5% significance level with a power of 95%) a sample size of 262 is necessary (using G*Power Version 3.2.1). F. B. Schuch et al. (2016) find a dropout rate of 17.2% in their meta‐analysis for depressed patients in exercise studies. Assuming a possible additional loss of 17% through overall missing data that is seen in studies using accelerometers (Helgadóttir et al., 2017), the sample size is enlarged to be 396, for practical reasons rounded up to 400 (40 per participating center, enables 34% of data loss by dropout or missing data).

#### 2.9.2 Statistical analyses

Accelerometer data are analyzed using Actigraph Software ActiLife 6.13.3. Minimal wearing time is eight hours a day, a PA measure of two complete days serving as minimal criteria to count as valid measurement.

Demographic and clinical characteristics of the study population are reported using descriptive statistics. Differences in baseline values between groups are assessed with chi‐squared tests or Fisher's exact test for nominal data and two‐sample *t*‐tests or Mann–Whitney *U* test for metric/ordinal data scales. Missing data will be imputed by multiple imputation techniques.

For assessing group differences in the primary outcome variables depression and PA, MANOVA is done with IBM SPSS Statistics. If baseline group differences occur (e.g., for age, gender, PA baseline level, comorbidity), we adjust for by analysis of covariance (MANCOVA). Furthermore, we report the percentage of participants in each group meeting criteria of WHO‐recommended PA to provide a benchmark to other PA studies. Likewise, we present remission and response rates for PI and TAU, respectively. Additional subgroup analyses are executed if necessary (e.g., concerning trial site, season of data collection, and medication). If MANOVA shows significant results, single ANOVA and discriminant analysis are executed to investigate the relationship between and the influences on the dependent variables.

Secondary data are analyzed by methods appropriate for the respective scale of the parameter and suitable for specific research questions. Results here are of exploratory nature.

### 2.10 Study approvals

The Ethics Commission of Charité Universitätsmedizin Berlin, Germany approves this trial (EA1/088/16). It also is registered (ClinicalTrials.gov Identifier: NCT02850341, registered January 08, 2016). Three amendments were further approved: (1) the extension of the local to a multicenter trial (May 2016), (2) an additional measurement of hand grip strength (May 2017), and (3) the addition of a reimbursement for the participation in the follow‐up‐measurement (June 2017).

### 2.11 Funding and sponsors

Responsible sponsor of this trial is Charité Universitätsmedizin Berlin (contact: Andreas Ströhle; andreas.stroehle@charite.de). This project is partly funded with 10,000 Euro by the “Robert‐Enke‐Stiftung.” Accelerometers are provided by Sport and Exercise Psychology, University of Potsdam, Potsdam, Germany. Additional equipment costs are covered by “Charité, Universitätsmedizin Berlin.” Funders and sponsor do not have any influence on planning or conducting of this trial and will not have any role in analysis, manuscript writing, or publication of this study.

## 3 RESULTS

### 3.1 Trial status and dissemination

Recruitment started in August 2016 and finished at the end of January 2020. Results are published, independently of magnitude or direction of found effects. Primary outcome paper is published first. Following additional dissemination via conference presentations and media is envisaged, likewise.

## 4 DISCUSSION

Our study is designed to test the efficacy of an adjunct PI in inpatient TAU. Since now, there is only little evidence available about flexible walking interventions in inpatient care for depressed patients. Hence, studies show promising results that this type of intervention might be well accepted especially by depressed patients (Busch et al., 2016) and effective in the reduction of symptoms.

Among the methodological constraints of this study are the absence of a nontreatment control group. However, we already have good evidence for the efficacy of inpatient depression treatment (Cuijpers et al., 2011; Mass, Backhaus, Hestermann, Balzer, & Szelies, 2019) and it would be unethical to deny treatment for severely depressed persons for such purposes. Furthermore, we cannot rule out the possibility that the mere act of measuring PA with accelerometers may have an influence on the participants' PA and PA motivation in both groups. We will bear this in mind when analyzing and interpreting our results. Another weak point may be that TAU already comprises PA interventions so that our effect to be found is exclusively due to the PI intervention, not to the efficacy of PA, overall. Yet, the use of an individually applicable add‐on intervention is the most suitable for a broad distribution in psychiatric clinics. It could be very easily adopted and implemented in routine inpatient treatment with the option of being continued even afterward. Physical exercise is effective in the treatment of depression but the remaining task is to develop effective PA interventions applicable into routine care. The wider aim of our trial is to promote the implementation of PA interventions in inpatient treatment of depression.

Strengths of this research project include:

1. First, we measure PA objectively (with some constraints: some types of PA cannot be registered correctly or at all like climbing or swimming and wear time cannot be controlled) and subjectively and provide an assessment of depression severity by blinded raters as well as subjectively reported depressive symptoms
2. Second, the population is generalizable, for being recruited in a naturalistic clinic field in different study centers. Center effects can be analyzed
3. Third, we will provide follow‐up data
4. Fourth, a unique aspect of our PA intervention is that it is personalized and adaptive concerning intensity, length, frequency, and personal circumstances and that is developed based on an empirically supported psychological background. Patients can execute it and continue it afterward in self‐management
5. Fifth, we take into account the effect of comorbid anxiety on our results

Our findings will contribute to the research field of exercise and depression in severely depressed patients and results will give opportunity for refining exercise‐related interventions in inpatient care.

## AUTHOR CONTRIBUTIONS

Andreas Ströhle conceived the study. Moritz Bruno Petzold and Andreas Ströhle initiated the study design and the PI and recruited participating centers. Moritz Bruno Petzold implemented the start of the trial and commented this protocol paper. Julia Große coordinated the study in the course and lead wrote this protocol paper. Ralf Brand advised on the selection of psychological constructs for the study and assisted in the writing of the manuscript. All authors read and approved the final manuscript. The authors thank Enrique Silva Cousino for providing technical support during the study.

## CONFLICT OF INTEREST

The authors declare no conflict of interest.
